# High Density Polyethylene Composites Reinforced with Hybrid Inorganic Fillers: Morphology, Mechanical and Thermal Expansion Performance 

**DOI:** 10.3390/ma6094122

**Published:** 2013-09-17

**Authors:** Runzhou Huang, Xinwu Xu, Sunyoung Lee, Yang Zhang, Birm-June Kim, Qinglin Wu

**Affiliations:** 1 College of Materials Science and Engineering, Nanjing Forestry University, Nanjing 210037, China; E-Mails: runzhouhuang@gmail.com (R.H); xucarpenter@yahoo.com.cn (X.X); yangzhang31@126.com (Y.Z); 2 Korea Forest Research Institute, Seoul 130-712, Korea; E-Mail: nararawood@forest.go.kr; 3 Department of Forest Products and Biotechnology, Kookmin University, Seoul 136-702, Korea; E-Mail: bkim3@tigers.lsu.edu; 4 School of Renewable Natural Resources, Louisiana State University Agricultural Center, Baton Rouge, LA 70803, USA

**Keywords:** glass fiber, talc, HDPE, composites, mechanical, thermal expansion

## Abstract

The effect of individual and combined talc and glass fibers (GFs) on mechanical and thermal expansion performance of the filled high density polyethylene (HDPE) composites was studied. Several published models were adapted to fit the measured tensile modulus and strength of various composite systems. It was shown that the use of silane-modified GFs had a much larger effect in improving mechanical properties and in reducing linear coefficient of thermal expansion (LCTE) values of filled composites, compared with the use of un-modified talc particles due to enhanced bonding to the matrix, larger aspect ratio, and fiber alignment for GFs. Mechanical properties and LCTE values of composites with combined talc and GF fillers varied with talc and GF ratio at a given total filler loading level. The use of a larger portion of GFs in the mix can lead to better composite performance, while the use of talc can help lower the composite costs and increase its recyclability. The use of 30 wt % combined filler seems necessary to control LCTE values of filled HDPE in the data value range generally reported for commercial wood plastic composites. Tensile modulus for talc-filled composite can be predicted with rule of mixture, while a PPA-based model can be used to predict the modulus and strength of GF-filled composites.

## 1. Introduction

As a new-generation green composite, co-extruded (core-shell structure) natural fibers reinforced polymer composites (NFPC) has been recently developed and used to enhance performance characteristics of composites. Co-extrusion technology has become one of the most advanced polymer processing technologies due to its unique capacity in creating a multi-layer composite with different complementary layer characteristics, and in making the properties of the final products highly “tunable”. In a core-shell structure wood polymer composites (WPC) system, the shell layer, made of thermoplastics unfilled or filled with minerals or natural fibers and other additives, plays a critical role in enhancing overall composite properties [1]. The shell layers with different material combinations, which have quite different properties, are, however, needed to achieve desired product performance.

Filled thermoplastics have been used widely as independent shell layer for co-extruded NFPC with core-shell structure. For example, it was demonstrated that a pure high-density polyethylene (HDPE) or pure polypropylene (PP) shell over a wood polymer composites (WPC) core reduced moisture uptake compare tonon-coextruded NFPC. However, the addition of a pure plastic shell with a relatively low modulus and large thermal expansion over a WPC core negatively affected overall composite modulus and thermal stability [1,2]. Investigations have also been done to develop a stabilized shell layer by blending HDPE and additives including a compatibilizer, a photostabilizer, and a nanosized TiO_2_ on the coextruded WPC, by using combined wood and mineral fillers [2], by using carbon nano-tube (CNT) in a shell layer, by using precipitated calcium carbonate (PCC) [3]. Further developments of filled materials as a more cost-effective shell layer for co-extruded WPC is still necessary.

Polymer composites reinforced with glass fibers (GFs) and talc may be achieved in the form of higher modulus and reduced material costs, yet accompanied with decreased strength and impact toughness [4]. Huang *et al.* [5,6], demonstrated the influence of varying shell moduli and thermal expansion coefficients of GF filled HDPE shells on the overall thermal expansion of co-extruded composites using a finite element model and described effect of the talc content for shell layers on mechanical and thermal expansion properties of core-shell structures WPC.

Hybrid filler reinforced composites form a complex system, and there is inadequate data available about phenomena behind the property changes due to the addition of particulate fillers to the fiber reinforced thermoplastic composites. Although individual classes of fillers or fibers can contribute some desirable properties as reinforcement filler for core-shell structure WPC, hybrid fillers have attracted much attention as the reinforced agents in the shell layer of core-shell structure WPC. The real interest in composites is in optimizing the different contributions from different types of fillers.

The objective of this study described in this paper was to investigate the effect of individual fillers (GF *vs.* talc) and combined fillers (talc and GF) on morphological, mechanical, and thermal expansion properties of the filled composites as potential shell material for coextrude NFPC/WPC. The result of this study can help provide a fundamental base for developing new functional applications of core-shell structure NFPC/WPC with hybrid fillers reinforced shells.

## 2. Results and Discussion

### 2.1. Composites with Individual Glass Fibers

#### 2.1.1. Morphology

**Figure 1:**
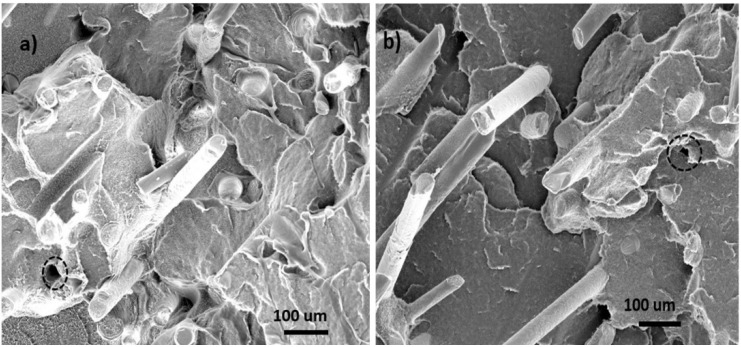
Scanning electron microscopy (SEM) micrographs of fractured surfaces of glass fiber (GF) filled high density polyethylene (HDPE) AD60 composites. (**a**) 10 wt % GF; (**b**) 30 wt % GF.

Scanning electron microscopy (SEM) was used to characterize the morphology of GF filled HDPE composites. Typical SEM micrographs for GF filled composites are shown in Figure 1 at 10 wt % and 30 wt % loading levels. Certain fiber pullout happened during the fracture process as indicated by the circular voids on the fracture plane. Fiber pullouts were observed on the surface of GF-filled HDPE composites (Figure 1) due to poor bonding of fiber to matrix leads to easy fiber pullout during the impact. Composites showed lower impact strength than neat HPDE due to insufficient fiber to matrix contact. This is consistent with low impact strength at 10 wt % and 30 wt % GF loaded compared with the pure HDPE.

Most glass fibers were aligned perpendicular to the fracture plane (*i.e.*, along the injection molding flow direction). Fiber breakage can contribute much less to energy than that the fiber pullouts in the net fractured energy [7]. A greater number of fiber pullouts can be observed on the fractured surface of a specimen with 30 wt % GF content than that of 10 wt % GF loaded. This was thought to be due fiber aggregation at the higher loading level in the composite matrix, which reduced their effective bonding.

#### 2.1.2. Tensile Properties

**Figure 2:**
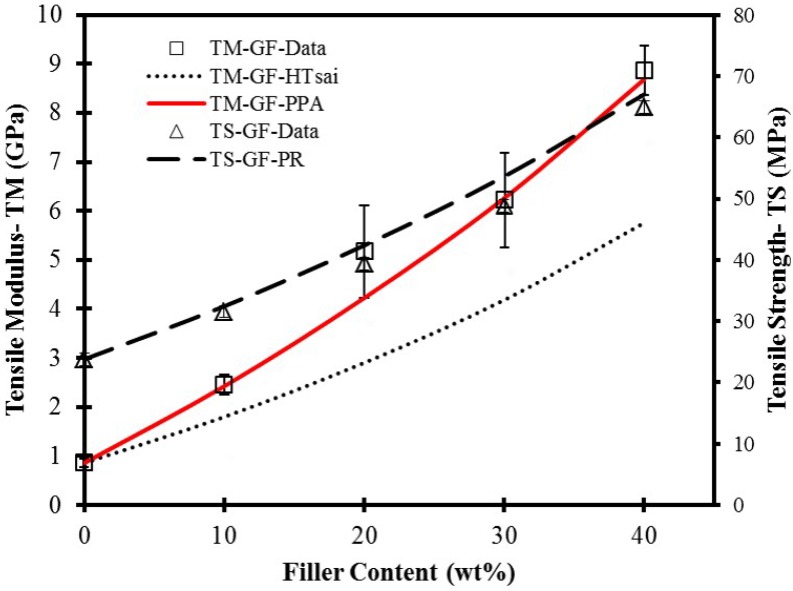
Tensile properties of GF-filled HDPE AD60 composites. Line showing predicted (PR) values with various models.

Tensile properties of neat HDPE and its composites with different glass fiber loading levels are summarized in Table 1 and plotted in Figure 2 (HDPE AD60 only). Tensile modulus for both resin systems increased with increase of the filler loading levels. For the neat HDPE AD60 and HDPE 6706, the tensile modulus was found to be 0.86 ± 0.09 GPa and 0.26 ± 0.02 GPa, respectively. The modulus increased to 8.87 ± 0.5 GPa at the 40% GF loading for HDPE AD60, and 5.37 ± 0.24 GPa at the 30% GF filling HDPE 6706. Apparently, GFs showed a better influence on tensile modulus of filled composites than that of pure HDPE matrix. This was due to a larger modulus value, a larger aspect ratio, and surface coupling treatment of the GFs to enhance their bonding to the matrix.

**Table 1:** Summary of mechanical properties of neat high density polyethylene (HDPE) and filled HDPE composites by individual glass fiber.

System	Filler Content (wt %) ^a^	Strength			Modulus	
		Tensile (MPa) ^b,c^	Flexural (MPa)	Impact (kJ/m^2^)	Tensile (GPa)	Flexural (GPa)
HDPE-6706/GF	0	18.9(0.2)A	19.76(0.39)A	8.12(0.21)A	0.26(0.02)A	0.73(0.05)A
	10	27.1(0.38)C	25.3(1.0)C	7.37(0.21)A	1.86(0.21)C	1.2(0.06)BC
	20	35.42(0.15)E	37.7(0.3)E	9.72(0.82)B	3.43(0.41)E	1.6(0.8)DE
	30	46.74(0.88)G	56.3(0.7)G	11.81(0.88)C	5.37(0.24)G	3.4(0.1)G
HDPE-AD60/GF	0	23.8(1.4)B	21.8(1.0)B	28.57(2.0)E	0.86(0.09)B	0.85(0.06)AB
	10	31.54(0.57)D	29.2(0.2)D	9.62(0.37)B	2.46(0.22)D	1.3(0.02)CD
	20	39.34(0.93)F	40.9(0.6)F	10.37(0.37)B	5.17(0.94)F	2.3(0.3)F
	30	48.80(0.50)H	57.8(0.7)H	11.94(0.16)C	6.22(0.97)G	3.6(0.08)G
	40 ^d^	64.92(0.34)I	85.9(1.6)I	14.55(0.34)D	8.87(0.51)H	5.8(0.2)H

^a^ The filler content was based on the total composite weight; ^b^ Mean values with the same capital letter for each property are not significantly different at the 5% significance level; ^c^ Numbers in the parenthesis are standard deviation based on five specimens; ^d^ Master batch of HDPE-GF blend.

There is a near linearly increasing trend of tensile strength for composite. The strength increased to 64.92 ± 0.34 MPa for GF-filled composites at the 40% GF loading level. Based on the statistical data analysis, GF filled HDPE composites showed the significantly enhanced behaviors of tensile strength. The bonding at the interface is a crucial parameter in determining the tensile strength.

The increasing trend of GF filled composites was due to surface coupling treatment of GFs helped develop a strong bond between the fiber and matrix, which avoid the stress concentration formed around the GF fiber in the stressed composites and allows the composite to bear more applied load.

For GF-filled composites, several theoretical models can be used to predict tensile properties of fiber composites in terms of the properties of the constituent materials [8]. The Halpi-Pagano micromechanical model [9], which has been utilized to predict the modulus of composites with randomly oriented short fibers [10,11], is given below:

(1) $$E_{\text{C}}^{\text{ran}}=\frac{3}{8}E_{L}+\frac{5}{8}E_{T}$$

where *E*_C_ is Young’s modulus of random fiber composites, *E*_L_ and *E*_T_ are longitudinal and transverse Young’s modulus, respectively, of corresponding uniaxial oriented discontinuous fiber composites. This equation is an averaging procedure for estimating elastic moduli of quasi-isotropic laminates. *E*_L _ and E_T_ can be estimated from the following Halpin-Tsai Equations [12,13].

(2) $$E_{L}=E_{m}\left[\frac{1+(2l/d)\eta_{L}\phi_{\text{f}}}{1-\eta_{L}\phi_{\text{f}}}\right]\text{ and }E_{T}=E_{\text{m}}\left[\frac{1+2\eta_{\text{T}}\phi_{\text{f}}}{1-\eta_{\text{T}}\phi_{\text{f}}}\right]$$

Among which the constant *η*_L_ and *η*_T_ are defined as:

(3) $$\eta_{\text{L}}=\frac{\left(E_{\text{f}}/E_{\text{m}}\right)-1}{\left(E_{\text{f}}/E_{\text{m}}\right)+\left(2l/d\right)}\text{ and }\eta_{\text{T}}=\frac{\left(E_{\text{f}}/E_{\text{m}}\right)-1}{\left(E_{\text{f}}/E_{\text{m}}\right)+2}$$

where *E*_f_ and *E*_m_ are Young’s moduli of the fiber and polymeric matrix, respectively; *ϕ*_f_ and *l/d* is volume fraction and aspect ratio of the fiber in the composites, respectively. The elastic modulus of short fiber composite can also be predicted based on paper physics approach-PPA [14,15]:

(4) $$E_{\text{C}}^{\text{PPA}}=\chi_{1}\chi_{2}E_{\text{f}}\phi_{\text{f}}+E_{\text{m}}(1-\phi_{\text{f}})$$

where *χ*_1_ and *χ*_2_ are, respectively, the fiber length and orientation factors for the composite elastic modulus.

To assess predictability of the tensile modulus of GF reinforced HDPE blends, experimental results were compared with calculated data from the above models (*i.e.*, Equations 1 and 4). The properties of the constituent materials of the composite used in the model prediction are *E*_f_ = 80 GPa, *E*_m_ = 0.86 GPa, density = 2.56 g/cm^3^ and *l*/*d* = 150 for glass fiber. Figure 2 shows comparisons of model predicted results with the experimental ones obtained from tensile tests as a function of filler loading level. As shown, the theoretical curves predicted by Equation 1 are far below the experimental data points for both filler systems. The behavior could be due to several reasons. First, the model assumes a perfect filler dispersion, while the presence of filler agglomeration may lead to underestimate of filler volume. Second, particle orientation may strongly influence the calculated Young’s modulus, while the model assumes a random fiber orientation. SEM micrographs (Figure 1) show significant alignment of the filler, especially for glass fibers. Equation 4 based on paper physics approach, PPA, was also used to calculate the tensile modulus. The calculated values based on a value of lumped parameter, *X*_1_ * *X*_2_, of 0.5 for glass fiber are also plotted in Figure 2. The predicted data are in a fairly good agreement with the experimental data.

The Nicolais and Nicodemo’s model [16] did not fit the GF-filled composite data at all due to the strong interfacial bonding between fiber and plastic matrix, which led to an increase of the composite tensile strength as fiber content increased.

Prediction of the tensile strength for randomly oriented short-fiber composites is a difficult task, and no universally accepted theory exists on this subject. The difficulty arises because the material’s ultimate strength in the case of composites is determined by the onset of fracture, and not via a yielding mechanism. Predictions are particularly difficult in the case of randomly oriented fibers, as cracks tend to propagate by fiber avoidance process as opposed to fiber pullout or fracture [17]. When predictions are attempted, they most frequently take the form:

(5) $$\sigma_{C}^{\text{PPA}}=X_{3}X_{4}\sigma_{\text{fu}}\phi_{\text{f}}+\sigma_{\text{mu}}\left(1-\phi_{\text{f}}\right)$$

where *σ*_fu_ is the fiber tensile strength; *σ*_mu_ is the tensile stress in the matrix at composite failure strain; *X*_3_ and *X*_4_ are the fiber-length and orientation-correction factors for tensile strength, respectively. The strength properties of the constituent materials of the composite used in the model prediction are *σ*_fu_ = 2400 MPa, *σ*_mu_ = 23.8 MPa, and a lumped parameter (*X*_3_·*X*_4_) of 0.1. Figure 2 shows a comparison of the calculated data from Equation 5 and experimental data for tensile strength of GF-based composites, which shows a reasonable agreement between the two data.

#### 2.1.3. Flexural Properties

Flexural properties of GF filled composites with varying amounts of each filler are shown in Table 1. Flexural modulus for GF filled composites exhibited an increasing trend with increased filler content. The neat HDPE AD60 had a flexural modulus of 0.85 ± 0.06 GPa while it was 5.8 ± 0.2 GPa for GF filled HDPE composites having 40 wt % of GF .The increase of flexural modulus was attributed to the enhanced interfacial interaction existed between the filler and matrix, which allowed the transmission of stress from HDPE to GF thereby improving the stiffness of the GF filled HDPE composite.

Based on the statistical data analysis, GF-filled composites showed a significant strength increase with increased GF loading level. At the 40% GF level, the strength was 2.29 times higher than that of the neat resin. Fiber alignment as shown in the SEM micrographs (Figure 1) played an important role in determining the flexural strength. The increased strength benefited from the uniaxially aligned GFs.

#### 2.1.4. Impact Strength

Table 1 shows test data of the notched Izod impact strength of GF filled composites at various leading levels. The neat AD60 resin had an impact strength of 28.57 ± 2.0 kJ/m^2^. The strength decreased significantly when the filler was added to the system. At the 10% filler loading level, the impact strength for GF filled composites are 9.62 ± 0.37 kJ/m^2^ and 6.03 ± 0.13 kJ/m^2^, respectively. For GF-filled composites, the impact strength increased with further increase of GF content beyond the 10 wt % level. As GFs are well bonded to the plastic matrix, the partially aligned GFs in the direction perpendicular to the impact force could help absorb the impact force imparted to the test samples. The enhanced impact strength at higher GF filling beyond 10 wt % due to GF fiber clustering in composite can be ruled out.

#### 2.1.5. Dynamic Mechanical Properties

**Figure 3:**
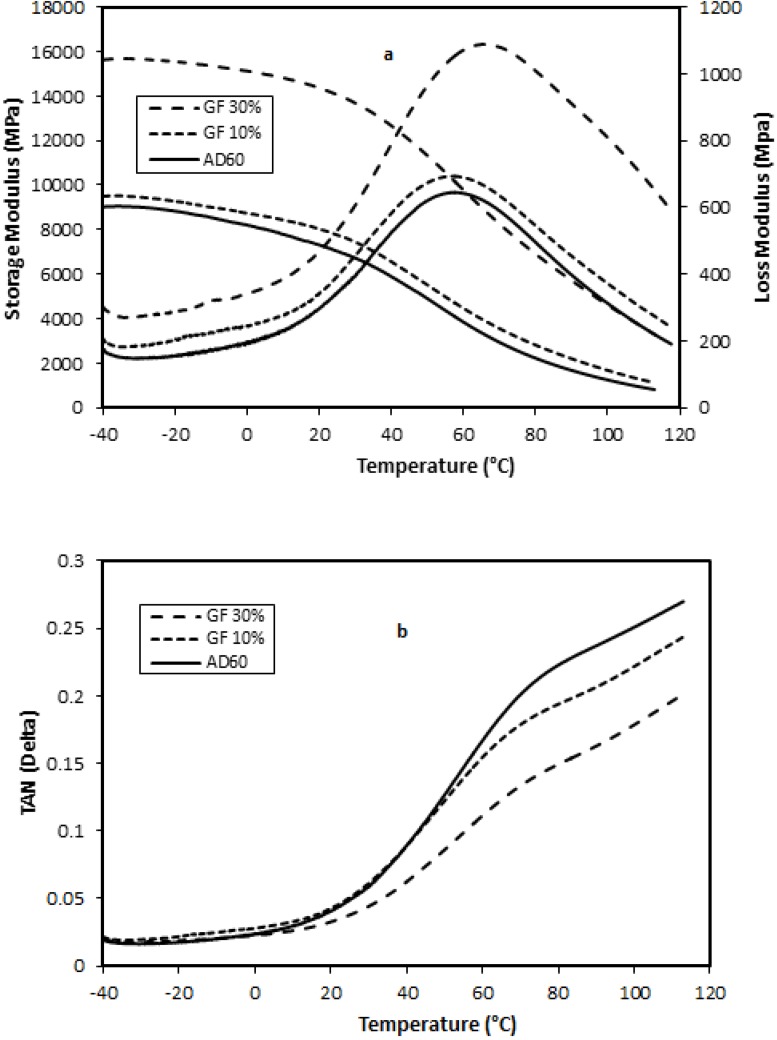
Effects of temperature level on storage modulus and loss modulus (**a**) and damping factor (**b**) of glass fiber-filled HDPE AD60 composites.

Storage Modulus (E′)—The effect of temperature on the storage modulus of GF-filled composites having 0%, 10%, and 30% loading levels is shown in Figure 3, respectively. A general trend of increase of the storage modulus with increased filler content in the composites was observed. Eʹ is more associated with the molecular elastic response of the composites, indicating the stiffness of the material. The increase of E′ with increased filler content was due to mechanical limitation posed by increasing filler concentration embedded in the viscoelastic matrix. The Eʹ decreased with temperature increase and converged to a narrow range at higher temperatures. The reduction of E′ with increasing temperature was due to the softening of the matrix and initiation of the relaxation process [18].

Loss Modulus (E″)—The loss modulus is a measure of the absorbed energy due to the relaxation and is associated with viscous response or the damping effect of the material. Figure 3 shows the effect of varying filler contents and temperature on loss modulus of the composites. Eʺ increased with the increased filler concentration and had a peak in the transition region around 50 °C. This relaxation peak was known as α-relaxation of HDPE [19], which was related to a complex multi-relaxation process associated with the molecular motion of the PE crystalline region [20,21]. The Eʺ at this relaxation temperature was markedly increased with the increase of filler loading level. The presence of a filler in the plastic resin reduced the flexibility of the material by introducing constraints on the segmental mobility of the polymer molecules. The α-relaxation peaks of GF-filled composites shifted to the higher temperature region as compared to neat HDPE resin.

Tan δ—The tan δ, damping factor, is a ratio of the loss modulus to the storage modulus. The parameter is independent of the material’s stiffness and is widely used to study viscoelastic response of the materials. Figure 3 shows tan δ curves of neat AD60 and filled composites with GF. For GF filled HDPE composite systems, tan δ curves had less distinctive α-relaxation process compared with the Eʺ data. For GF-filled composites, the damping curves shifted toward lower values as the filler content level increased. This indicates that the damping effect reduced with the increased filler content in the matrix. The result suggested that certain degree of interfacial bonding existed between the fillers and matrix in GF-filled composites. The higher GF levels induced a better fiber packing in the matrix and resulted in more efficient stress transfer from the resin matrix to the fibers, leading to decreased damping effect. Composites showed interfacial adhesion, leading to change in the damping effect.

#### 2.1.6. Thermal Expansion Properties

**Figure 4:**
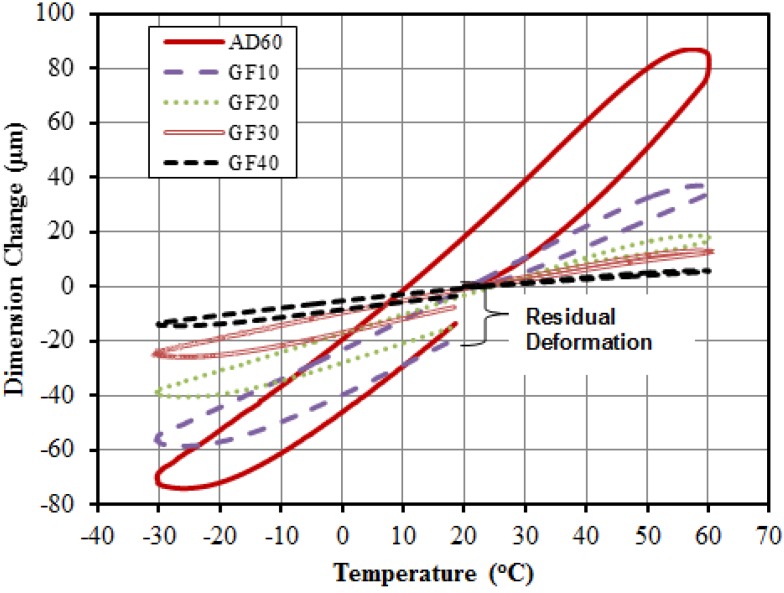
Typical dimension change-temperature history for glass fiber filled HDPE AD60 composite systems.

Typical dimension change data in relation with temperature for the composite system is shown in Figure 4 for neat AD60 resin and GF filled systems. The sample dimension increased as the temperature increased, and decreased as temperature decreased. The linear coefficient of thermal expansion, LCTE, is represented by the slope of the linear portion of the curve. Neat plastic had an obvious larger dimension change than that from filled composites for a given temperature change. Residual deformation is seen for the neat plastic (AD60) at the end of the heating cycle. For the GF-system, the residual deformation was significantly lowered at the two higher GF loading levels (*i.e.*, 30%, and 40%).

The measured LCTE values over the three temperature ranges (*i.e.*, heating: 20–>60 °C, cooling: 60–>−30 °C); and heating: −30–>20 °C) are summarized in Table 2 as a function of filler content levels for GF filled composites. The first heating cycle led to the largest LCTE values at each given filler content levels. LCTE decreased significantly as the GF content was added to the system. At the 40% GF level, the LCTE values are respectively, 11.1, 17.1, and 19.7 × 10^−6^/°C from 199.1, 162.7, 137.8 × 10^−6^/°C for the three temperature ranges due to GF causes a mechanical restrain on HDPE chain opening during the temperature change. The values are much smaller than the reported LCTE values of commercial WPC. Thus, the use of chemically modified GFs is more effective in controlling thermal expansion behavior of the filled plastics composite. The system can be used as an effective shell layer in co-extruded NFPC/WPC to control overall thermal expansion properties of the composites [5].

**Table 2:** Summary of thermal expansion properties of virgin HDPE and filled HDPE composites with individual glass fibers.

System	Filler content (wt %)	Linear Coefficient of Thermal Expansion (LCTE) (10^−6^/°C) ^a,b,c^		
		20→60 °C	60→−30 °C	−30→20 °C
HDPE 6706/GF	0	203.9(3.0)G	164.8(6.3)G	143.8(4.8)I
	10	71.1(0.4)E	79.7(1.4)E	82.5(1.1)F
	20	32.0(1.1)C	41.6(3.8)C	50.9(0.8)D
	30	19.8(0.7)B	28.6(1.5)B	34.1(1.1)C
HDPE AD60/GF	0	199.1(2.8)G	162.7(2.5)G	137.8(1.0)H
	10	75.1(0.5)F	87.6(3.1)F	87.8(0.7)G
	20	39.6(2.2)D	55.1(0.1)D	57.8(0.7)E
	30	22.4(0.3)B	33.1(1.8)B	33.9(0.3)B
	40	11.1(0.2)A	17.1(0.2)A	19.7(0.2)A

^a^ The content of each filler was based on the total composite weight; ^b^ Mean values with the same capital letter for each property are not significantly different at the 5% significance level; ^c^ Numbers in the parenthesis are standard deviation based on five specimens.

### 2.2. Composites with Combined Talc and Glass Fiber Fillers

#### 2.2.1. Morphology

**Figure 5:**
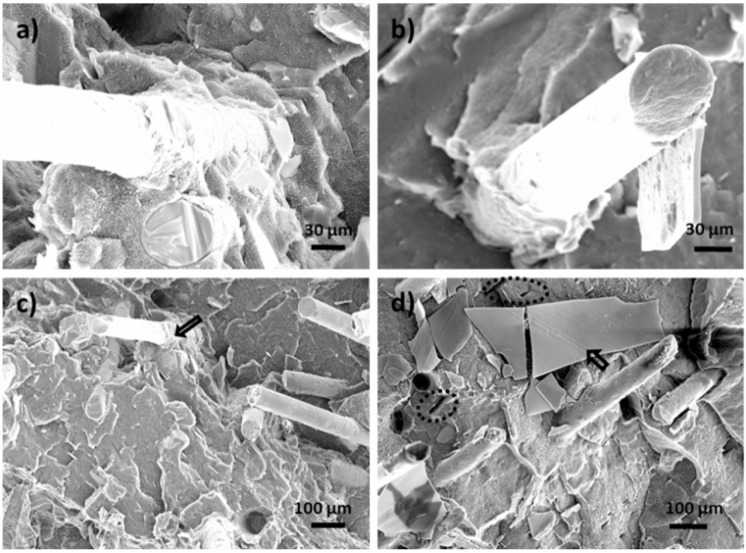
SEM micrograph of fractured surfaces of composites with combined fillers. Upper two charts (**a**,**b**) 10 wt % filer(talc/glass fiber = 1:2); and Lower two charts (**c**,**d**) 30 wt % filler (talc/glass fiber = 1:2).

Typical SEM micrographs for composites filled with the combined talc and glass fibers (1:2 mixing ratio) are shown in Figure 5 (a and b: 10% loading level; c and d: 30% loading level). Similar to the morphology of the individual filler systems, the plate talc particles were oriented both perpendicular and parallel to the fracture plane. The perpendicular-to-the-fracture-plane orientation of the talc particles is displayed by several line-type pull-out voids in Figure 5d. Most GFs are perpendicular to the fracture plane. The GFs bonded well to the plastic matrix, while there was little bonding between talc and plastic matrix as indicated by the arrows in Figure 5c,d.

#### 2.2.2. Mechanical Properties

**Figure 6:**
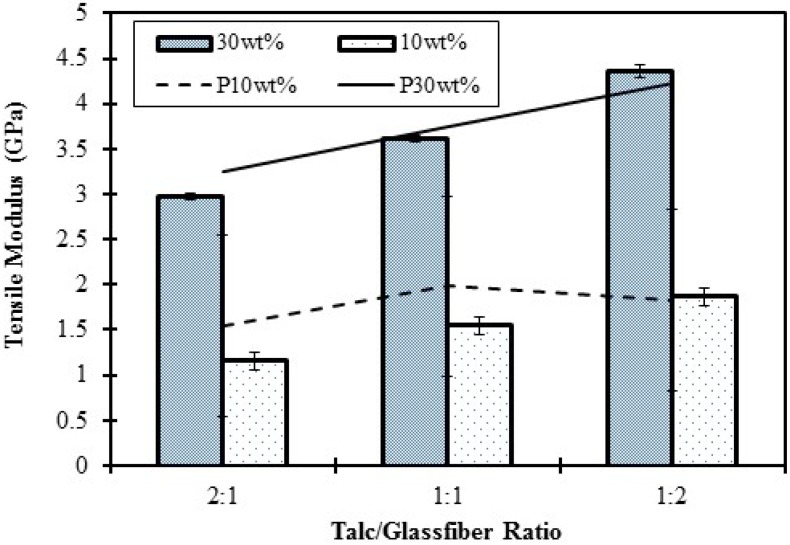
Tensile modulus of composites filled with combined glass fiber and talc fillers. Lines in the upper chart showing predicted values.

Mechanical properties of neat HDPE and composites with hybrid talc and GF fillers are shown in Table 3. Tensile modulus *vs.* talc/glass fiber ratios are plotted in Figure 6. Here, both HDPE 6706 and HDPE AD60 were used to generate a mixed blend (50/50 wt %) as the matrix. For the two neat resins, mechanical properties of HDPE AD60 are higher than those of HDPE 6706. Increase of combined fillers content, from 10% to 30%, led to increased mechanical properties. At a fixed filler loading level, the increase of GF portion of the filler led to increased strength and modulus of composites, especially at 30% loading level.

**Table 3:** Summary of mechanical properties of neat HDPE and filled HDPE composites with combined fillers.

System	Filler content (wt%) ^a^	Talc/GF Ratio	Strength			Modulus	
			Tensile (MPa) ^b,c^	Flexural (MPa)	Impact (kJ/m^2^)	Tensile (GPa)	Flexural (GPa)
HDPE6706	0	0	18.9(0.2)A	19.8(0.4)A	8.12(0.21)E	0.26(0.02)A	0.73(0.05)A
HDPEAD60	0	0	23.8(1.4)C	21.8(1.0)B	28.57(2.0)F	0.86(0.09)B	0.85(0.06)B
HDPEAD60/ HDPE6706/ Talc/GF	30	2:1	27.7(0.48)D	33.7(1.4)D	5.77(0.59)A	2.9(0.13)D	2.0(0.20)E
		1:1	31.9(0.41)E	38.8(0.3)E	6.35(0.22)AB	3.6(0.39)E	2.4(0.06)F
		1:2	36.8(0.63)F	44.7(0.6)F	7.80(0.36)DE	4.4(0.33)F	2.8(0.07)G
	10	2:1	21.8(0.22)B	26.4(0.3)C	5.43(0.35)A	1.2(0.15)B	1.2(0.01)C
		1:1	22.6(0.36)B	26.8(0.8)C	6.12(0.30)AB	1.6(0.23)C	1.3(0.05)C
		1:2	24.6(0.23)C	27.1(0.6)C	7.03(0.11)CD	1.9(0.15)C	1.6(0.03)D

^a^ The filler content was based on the total composite weight; ^b^ Mean values with the same capital letter for each property are not significantly different at the 5% significance level; ^c^ Numbers in the parenthesis are standard deviation based on five specimens.

An attempt was made to use the paper physics approach to predict tensile modulus of the three component system (*i.e.*, talc, GF, and resin).

(6) $$E_{\text{C}}\;=\;X_{\text{GF}}E_{\text{GF}}\Phi_{\text{GF}}\;+\;X_{\text{Talc}}E_{\text{Talc}}\Phi_{\text{Talc}}\;+\;E_{\text{m}}(1\;-\;\Phi_{\text{GF}}\;-\;\Phi_{\text{Talc}})$$

where *X*_GF_ and *X*_talc_ are, respectively, the combined length and orientation factors for GF and talc fillers. Figure 4 showed a comparison between predicted and experimental values of the tensile moduli. With *X*_GF_ = 0.4 and *X*_talc_ = 1.0, the predicted values from Equation 6 showed a reasonable agreement with experimental data.

**Figure 7:**
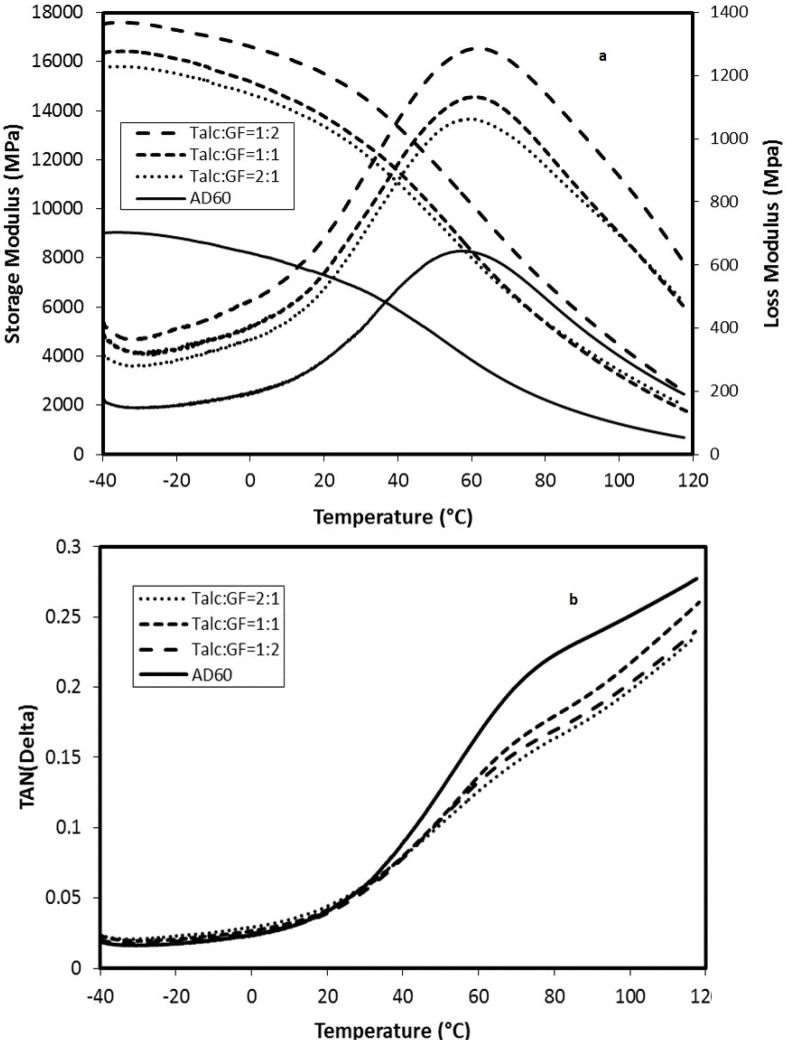
Effects of temperature level on storage modulus and loss modulus (**a**) and damping factor; (**b**) of HDPE AD60 composites filled with combined GF and talc fillers at the 30 wt %.

Dynamic mechanical results (Figure 7) supported flexural modulus trend listed in Table 4 base on the Eʹ and Eʺ values. The temperature range of a-relaxation shifted between 50 and 62 °C for both filler weight contents. The comparison between Table 3 and Table 4 showed that modulus and strength of composites, modified by combined filler, were sight lower than that of GF alone. The tan δ was decreased with increasing filler content. The 30 wt % combined filler reinforced HDPE composites showed higher tan δ than that of 10 wt % combined filler, and the tan δ values of the combined filler reinforced composite were seen to be lower than that of the pure HDPE matrix. The reason of the above those result was closely similar with that of individual filler.

**Table 4:** Summary of thermal expansion properties of virgin HDPE and filled HDPE Composites with combined glass fiber and talc filler.

System		Filler content (wt %) ^a^		Talc/GF ratio		LCTE (10^−6^/°C) ^a,b,c^			
						20→60 °C	60→30 °C	−30→20 °C	
HDPE6706		0		0		203.9(3.0)G	164.8(6.3)G	143.8(4.8)G	
HDPEAD60		0		0		199.1(2.8)G	162.7(2.5)F	137.8(1.0)F	
HDPE AD60/ HDPE6706/ Talc/GF		10		2:1		144.9(3.4)F	123.2(8.4)E	113.5(2.3)E	
				1:1		117.7(0.9)D	111.3(2.1)D	105.1(0.9)D	
				1:2		124.0(0.7)E	114.2(3.2)D	107.0(0.5)D	
		30		2:1		75.5(2.7)C	67.1(2.5)C	66.3(0.7)C	
				1:1		48.2(0.7)A	47.8(1.3)A	52.5(0.3)A	
				1:2		54.6(0.5)B	53.8(2.2)B	54.8(0.6)B	

^a^ The content of each filler was based on the total composite weight; ^b^ Mean values with the same capital letter for each property are not significantly different at the 5% significance level; ^c^ Numbers in the parenthesis are standard deviations based on five specimens.

#### 2.2.3. Thermal Expansion

**Figure 8:**
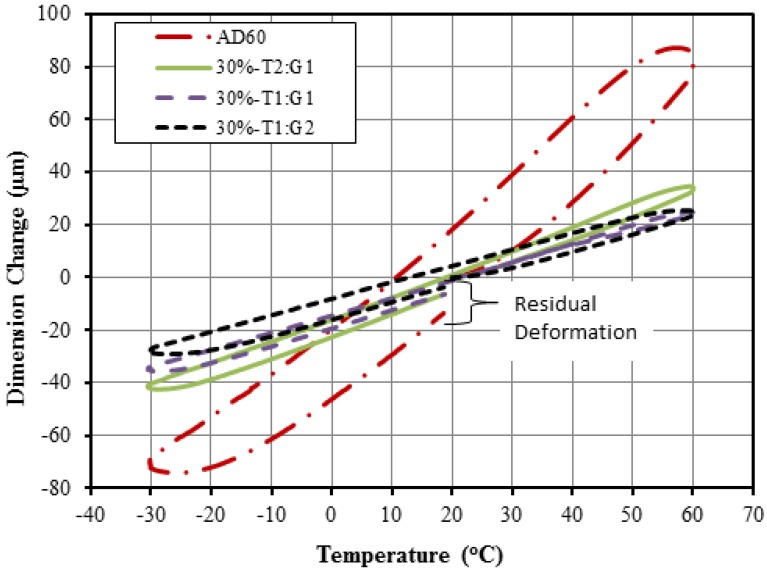
Typical dimension change-temperature history for HDPE AD60 composite filled with combined glassfiber and talc fillers at the 30 wt % loading level.

Typical dimension change and temperature relationship curves for the combined filler systems are shown in Figure 8 in comparison with neat resin data values. LCTE value is represented by the slope of the linear portion of the curve Pure HDPE had an obvious larger dimension change than that from filled composites for a given temperature range. As the GF content increased the curves turned more clockwise toward reduced dimension changes. Residual deformation is seen for the neat HDPE at the end of the heating cycle. The use of combined fillers at the 30% loading level largely reduced residual deformation of the filled composites.

Table 4 lists of measured LCTE data for the composites with combined fillers. LCTE values at the three talc/GF ratios and two total loading levels was showed difference trend. As the total filler level (*i.e.*, 10% *vs.* 30%) and GF portion of the alc/GF fillers (*i.e.*, 2:1, 1:1, and 1:2) increased, the LETC values decreased. The total of 10% filler loading led to the LCTE values of 124.0, 117.7, and 115.0 × 10^−6^/°C at talc/GF ratios of 2:1, 1:1, and 1:2, respectively. The use of the 30% combined fillers reduced the LCTE values to 75.5, 48.2, and 54.6 × 10^−6^/°C at talc/GF ratios of 2:1, 1:1, and 1:2, respectively. The reason what was closely similar with that of individual filler caused the above those results.

## 3. Experimental Section

### 3.1. Raw Material and Experimental Design

SGF reinforced HDPE pellets were provided by RTP Co. (Winona, MN, USA). The material was of the type RTP 707 CC UV Natural, a SGF reinforced HDPE with glass fiber content of 40 wt %. The fiber diameter was 0.014 mm and fiber length was 4 mm prior to compounding. Fibers were sized with a silane-based solution before compounding. UV and coupling agent were added to the system during compounding. Talc was provided by Fiber Glast Development Corp. (Brookville, OH, USA). Two types of neat HDPE were provided by ExxonMobil Chemical Co. (Houston, TX, USA).

Experiment design included two factorial experiments. The first experiment was to investigate the effect of individual filler consisting of six blends covering one filler (glass fiber) and four loading rates (40, 30 , 20, and 10 wt % of total composite weight for glass fiber pallets filler reinforced HDPE AD60 and HDPE 6706, respectively). The second experiment was designed to study the effect of combined polymer and combined fillers system (HDPE AD60/HDPE 6706/Talc/glass fiber pallets), consisting of six blends covering two filler weight contents (10 and 30 wt %) and three talc/glass fiber ratios (2:1, 1:1, and 1:2). The pure HDPE (HDPEAD60 and HDPE 6706) and glass fiber pallets (40 wt % of total composite weight for glass fiber reinforced HDPE AD60) were used as a control.

### 3.2. Sample Preparation

Melt compounding was performed using an intermesh, counter-rotating Brabender twin-screw extruder (Brabender Instruments, Hackensack, NJ, USA) with a screw speed of 40 rpm. The temperature profile of barrels ranged from 150 to 175 °C. The extrudates were air-cooled and then pelletized into granules. The granules were injection-molded into standard mechanical test specimens using a Batenfeld Plus 35 injection molding machine (Batenfeld, NJ). The injection temperatures were 190 and 180 °C for HDPE/filler composites and virgin HDPE, respectively. All specimens were then conditioned for 72 h at a temperature of 23 ± 2 °C and a relative humidity of 50% ± 5% for later characterization.

### 3.3. Characterization and Data Analysis

Morphology Analysis. The scanning electron micrographs was taken by Field Emission Scanning Electron Microscopy (FESEM, FEI Quanta TM 3D FEG Dual Beam SEM/FIB, Hillsboro, OR, USA) and used to analyze the morphology of fractured surfaces of select samples. Prior to the microscopy, samples were frozen with liquid nitrogen for 10 min, and then were impact-fractured. The fractured surfaces of the specimens were coated with gold t to avoid electrical charging. The acceleration voltage used was 10.0 KV in SEM analysis.

Static Strength Property. Flexural testing was done with specimens of 80 mm × 13 mm × 3 mm under three-point bending using an Instron 5582 testing machine (Instron, Norwood, MA) following ASTM D790. A crosshead speed of 1.37 mm/min and a span length of 50 mm were used for test. Tensile testing was done with Type-I dumbbell-shape tensile specimens having a dimension of 165 mm × 13 mm × 3 mm using the same Instron machine according to ASTM D638. A crosshead speed of 5 mm/min and an extensometer with a gage length of 25 mm for strain measurement were used for the test. Notched Izod impact strength was determined from specimens of 63.5 mm × 12.7 mm × 3 mm in size using a Tinius Olsen Mode 1892 impact tester (Tinius Olsen, Horsham, PA, USA) according to ASTM D256. The notch angle of 45o and “V”-type notch depth of about 2.5 mm were used for the test. Five specimens were taken for each test and average data along with corresponding standard deviation were reported.

Dynamic Mechanical Analysis (DMA). The storage modulus, loss modulus, and tan δ of neat resin and its composites were evaluated using a DMA Q800 (TA Instruments Inc., New Castle, PA, USA) with samples of 59.8 (length) mm × 12.7 (width) mm × 3.2 (thickness) mm. The specimens were heated from −40 to 120 °C with a heating rate of 5 °C/min. A dual cantilever mode was used to test all specimens at a fixed frequency of 1 Hz.

Thermal Expansion. Thermal expansion samples were machined with a miniature table saw along the long direction of injection molded samples with dimension of 5.1 mm × 12.7 mm × 1.6 mm. Special attention was made to ensure that the sample ends are parallel. The test was done with a TA Q400 ThermoMechanical Analyzer, TMA .The sample was placed on a quartz base and an extension quartz probe was then placed on the top surface of the sample. A loading of 5 g force was applied to the probe to ensure the proper contact of the probe and the sample. The change in the length of the sample with temperature was measured using a linear variable differential transformer (LVDT) with a sensitivity of ±0.02 μm. The length and temperature data were recorded and analyzed with TA’s Universal Analysis software. All tests were done with three heating cycles—(1): 20 °C to 60 °C; (2) 60 °C to –30 °C; and (3): –30 °C to 20 °C. The heating and cooling rates were kept constant at 5 °C/min.

### 3.4. Statistical Data Analysis.

Duncan’s multiple range tests for pair-wise comparisons were used to test the effect of various treatments on measured properties using Statistical Analysis Software SPSS 20.0 [22]. Statistical ranking at the 5% significance level was provided among the treatments for each property.

## 4. Conclusions 

The effect of individual and combined talc and short glass fibers on mechanical and thermal performance of the filled HDPE composites was studied. Several published models were adapted to fit the measured tensile moduli and strength of various composite systems. The following conclusions can be drawn from the study.

(1) The use of silane-modified short GFs had a much larger effect in improving mechanical properties and in reducing LCTE values of filled composites compared with the use of talc due to enhanced bonding to the matrix, larger aspect ratio and fiber alignment for GFs.
(2) Mechanical properties and LCTE values of composites with combined talc and GF fillers varied with talc and GF ratio at a given total filler loading level. The use of a larger portion of GFs in the mix can lead to better composite performance, while the use of talc can help lower the composite costs and increase its recyclability. The use of 30 wt % combined filler seems necessary to control LCTE values of filled HDPE in the data value range generally reported for commercial wood plastic composites.
(3) Tensile modulus for talc-filled composite can be predicted with rule of mixture, while a PPA-based model can be used to predict the modulus and strength of GF-filled composites.
(4) The material developed can be used as an effective shell layer in co-extruded NFPCs/WPCs to enhance their performance properties.
